# Mouse Strain-Dependent Difference Toward the *Staphylococcus aureus* Allergen Serine Protease-Like Protein D Reveals a Novel Regulator of IL-33

**DOI:** 10.3389/fimmu.2020.582044

**Published:** 2020-09-25

**Authors:** Andrea R. Teufelberger, Sharon Van Nevel, Paco Hulpiau, Maria Nordengrün, Savvas N. Savvides, Sarah De Graeve, Srinivas Akula, Gabriele Holtappels, Natalie De Ruyck, Wim Declercq, Peter Vandenabeele, Lars Hellman, Barbara M. Bröker, Dmitri V. Krysko, Claus Bachert, Olga Krysko

**Affiliations:** ^1^Upper Airways Research Laboratory, Department of Otorhinolaryngology, Ghent University, Ghent, Belgium; ^2^Department of Biomedical Molecular Biology, Ghent University, Ghent, Belgium; ^3^Howest, University College West Flanders, Bruges, Belgium; ^4^Department of Immunology, University Medicine Greifswald, Greifswald, Germany; ^5^Unit for Structural Biology, VIB Center for Inflammation Research, Ghent, Belgium; ^6^Department of Biochemistry and Microbiology, Ghent University, Ghent, Belgium; ^7^The Biomedical Center, Department of Cell and Molecular Biology, Uppsala University, Uppsala, Sweden; ^8^Molecular Signaling and Cell Death Unit, VIB Center for Inflammation Research, Ghent, Belgium; ^9^Cell Death Investigation and Therapy Laboratory, Department of Regeneration and Repair, Ghent University, Ghent, Belgium; ^10^Cancer Research Institute Ghent, Ghent, Belgium; ^11^Institute of Biology and Biomedicine, National Research Lobachevsky State University of Nizhni Novgorod, Nizhny Novgorod, Russia; ^12^International Airway Research Center, First Affiliated Hospital, Sun Yat-sen University, Guangzhou, China; ^13^Department of Ear, Nose and Throat Diseases, Karolinska University Hospital, Stockholm, Sweden

**Keywords:** allergy, asthma, IL-33, *S. aureus*, SplD, type 2 immunity

## Abstract

*Staphylococcus aureus* (*S. aureus)* can secrete a broad range of virulence factors, among which staphylococcal serine protease-like proteins (Spls) have been identified as bacterial allergens. The *S. aureus* allergen serine protease-like protein D (SplD) induces allergic asthma in C57BL/6J mice through the IL-33/ST2 signaling axis. Analysis of C57BL/6J, C57BL/6N, CBA, DBA/2, and BALB/c mice treated with intratracheal applications of SplD allowed us to identify a frameshift mutation in the serine (or cysteine) peptidase inhibitor, clade A, and member 3I (S*erpina3i*) causing a truncated form of SERPINA3I in BALB/c, CBA, and DBA/2 mice. IL-33 is a key mediator of SplD-induced immunity and can be processed by proteases leading to its activation or degradation. Full-length SERPINA3I inhibits IL-33 degradation *in vivo* in the lungs of SplD-treated BALB/c mice and *in vitro* by direct inhibition of mMCP-4. Collectively, our results establish SERPINA3I as a regulator of IL-33 in the lungs following exposure to the bacterial allergen SplD, and that the asthma phenotypes of mouse strains may be strongly influenced by the observed frameshift mutation in S*erpina3i*. The analysis of this protease-serpin interaction network might help to identify predictive biomarkers for type-2 biased airway disease in individuals colonized by *S. aureus*.

## Introduction

Several type 2 airway diseases, such as allergic rhinitis, chronic rhinosinusitis with nasal polyps, and allergic asthma are associated with nasal colonization by *Staphylococcus aureus* (*S. aureus*) (1, 2). *S. aureus* can secrete a wide range of virulence factors, among which staphylococcal serine protease-like proteins (Spls) have been identified as bacterial allergens and inducers of allergic asthma in C57BL/6J mice by activating the IL-33/ST2 signaling axis (3, 4). Spl-specific IgE levels are increased in asthmatic patients compared to healthy controls, and detectable amounts of Spls have been found in nasal polyp tissue (3, 4). Strikingly, 25–30% of the general population are persistently colonized with *S. aureus* and up to 69% are intermittent or occasional *S. aureus* carriers (5). Even though there is strong evidence for *S. aureus* being an inducer, enhancer, and driver of chronic inflammatory airway diseases (3, 6–9), it is not understood why not all persistent *S. aureus* carriers develop a chronic inflammatory response toward it. Since *S. aureus* exposure on its own cannot explain the pathogenesis, a genetic predisposition might be underlying the response toward *S. aureus*.

IL-33 is an innate cytokine of the IL-1 family, which is expressed in epithelial, endothelial (10), and smooth muscle cells (11), as well as in fibroblasts, activated mast cells (12), and dendritic cells (13), and released upon allergen exposure or during necrosis (14, 15). IL-33 mediates Th2 cytokine production in innate lymphoid cells type 2 (ILC2s), Th2 cells, and invariant natural killer T cells by binding to its membrane bound receptor ST2 (13, 16–19). Like other IL-1 cytokine family members, the cytokine activity of IL-33 can be regulated by proteolytic cleavage (20). When it is cleaved by endogenous proteases or proteolytic allergens in the cleavage/activation domain, the cytokine activity of IL-33 is increased (21–25). Inactivation of IL-33 occurs by cleavage in the IL-1-like cytokine domain through endogenous proteases such as the human neutrophil proteinase 3, the human mast cell chymase and the murine chymase mouse mast cell protease 4 (mMCP-4), or by the caspases 3 and 7 in humans and mice (26–29).

Endogenous protease activity is, in turn, under tight regulation of serine protease inhibitors, such as serpins. Serpins inhibit proteases with their C-terminal reactive center loop (RCL). Upon cleavage in the RCL, they form SDS-stable complexes with their target proteases by covalent binding (30). The murine *Serpina3i* is expressed at low levels in the lungs, thymus, and spleen during homeostasis (31).

In the present study we have identified SERPINA3I as a novel regulator of IL-33 processing in a murine asthma model. Interestingly, BALB/c mice carry a frameshift mutation in the S*erpina3i* gene, leading to faster cleavage of IL-33 upon serine protease-like protein D (SplD) exposure. This observation may explain the lack of a type 2 inflammatory response upon SplD challenge of the lungs. Our results suggest that SERPINA3I is important for controlling inflammatory reactions in mice.

The aim of this study was to find the underlying genetic difference between BALB/c and C57BL/6J mice leading to the different response toward SplD.

## Materials and Methods

### Recombinant Protein Production

Recombinant SplD was produced in the *Bacillu*s *subtilis* strain 6051HGW LS8P-D, which lacks the proprietary proteases WprA, Epr, Bpr, NprB, NprE, Vpr, Mpr, and AprE, as previously described in detail (3, 4). SplD was purified from the cell-free supernatants by means of ion-exchange chromatography on an SP Sepharose Fast Flow column (GE Healthcare, Fairfield, CT, United States), followed by a 2-step purification with centrifugal filter units (Amicon Ultra 30K/10K, Merck Millipore, Billerica, MA, United States). The buffer was exchanged to PBS and the quality of the native SplD preparation was verified by using SDS-PAGE. Recombinant full-length, C-terminal HIS tagged SERPINA3I (isoform X3; NCBI reference sequence: XP_017170642.1) was produced in HEK293 cells by ProSpec-Tany Technogene Ltd. (Rehovot, Israel). The purified protein was endotoxin low (<0.01 EU/μg) and provided in PBS. Proteolytically active mMCP-4 was produced and activated essentially as previously described in Andersson et al. (32).

### Mice and Treatment Protocols

Female C57BL/6JRj (C57BL/6J), C57BL/6NRj (C57BL/6N), BALB/cJRj (BALB/c), CBA/JRj (CBA), and DBA/2 JRj (DBA/2) wild-type mice (Janvier Labs, Saint-Berthevin Cedex, France) were 6–8 weeks old during the treatment protocols. Animals were kept under standard conditions, in the individually ventilated (IVC) cages, 12 h light/dark cycle, access to food and water *ad libitum*. All animal experiments were approved by the local ethics committees of Ghent University.

For the short-term treatment, mice were treated with an intratracheal (i.t.) application of either 50 μL PBS or 50 μg SplD in 50 μL PBS once and euthanized 6 or 12 h after the single treatment. For the long-term sensitization protocol, C57BL/6J, C57BL/6N, CBA, DBA/2, and BALB/c mice were treated with six i.t. applications of either 50 μL PBS (Gibco, Erembodegem, Belgium) or 45 μg SplD in 50 μL PBS with one additional application of 50 μL PBS every 2 days. Additional groups of BALB/c mice were treated with either 50 μL PBS and 200 ng murine IL-33 (Peprotech, Rocky Hill, NJ, United States) in 50 μL PBS or with 45 μg SplD in combination with 200 ng of IL-33 in 50 μL PBS (SplD + IL-33) in the same manner as the other treatment groups. All mice underwent light anesthesia by inhalation of isoflurane/air (Ecuphar, Oostkamp, Belgium) during every application. 48 h after the last i.t. application, mice were euthanized with an intraperitoneal injection of 100 μL nembutal (Ceva Santé Animale, Libourne, France). For the reconstitution of SERPINA3I BALB/c, mice received 50 μg SplD in combination with 5 μg SERPINA3I (SplD + SERPINA3I), or sole 5 μg SERPINA3I, and were euthanized after 12 h. Serum, bronchoalveolar lavage fluid (BALF) and lungs were collected and processed as previously described (4). All experimental protocols are summarized in Supplementary Table 2.

### Organ Processing

Blood was collected in EDTA Microvettes 200Z (Sarstedt, Nümbrecht, Germany) and centrifuged for 10 min at 3000 rpm. Serum was collected and stored at -20°C before further analysis. BALF was collected by rinsing the airways three times with 0.3 mL of 1% bovine serum albumin (BSA; Sigma Aldrich, Diegem, Belgium) in PBS with complete protease inhibitor cocktail (Roche Diagnostics, Anderlecht, Belgium) and twice with 1 mL of 0.2% EDTA (Sigma-Aldrich) in PBS and kept at 4°C. Red blood cells were lysed for 2 min with VersaLyse buffer (Beckman Coulter, Krefeld, Germany), and BALF cells were used for flow cytometry analysis. The left lobe of the perfused lungs was fixed with 10% formalin (Sigma-Aldrich) for paraffin embedding, while the right lobes were either snap-frozen or minced and digested with 1 mg/mL collagenase type II (Worthington Biochemical, Lakewood, NJ, United States) for flow cytometry analysis. Snap-frozen lungs were either used for protein or RNA extraction. For protein extraction, they were homogenized by using the Tissue Lyser LT (Qiagen, Antwerp, Belgium) for 2 min at 50 oscillations/s with 10 times more w/v T-Per Tissue Protein Extraction Reagent (Thermo Fisher Scientific) with HALT protease inhibitor cocktail kit (Thermo Fisher Scientific). After 10 min of centrifugation at 3000 rpm, supernatants were collected and repeatedly centrifuged at 15,000 rpm for three in each. The total protein concentration of the lung homogenates was determined with the Bio-Rad Protein assay (Bio-Rad Laboratories, Hercules, CA, United States) in the collected supernatants.

### Western Blotting

Thirty μg protein of lung homogenates were denatured for 10 min at 95°C with sample buffer under reducing conditions (sample buffer: Laemmli buffer with 5% β-mercapto-ethanol and 0.25% Bromophenol blue) and loaded on 4–15% mini-PROTEAN TGX Stain-Free Protein gels (Bio-Rad), separated by means of SDS-PAGE and transferred to a nitrocellulose membrane (Bio-Rad). For Western Blotting, mouse IL-33 antigen affinity-purified polyclonal goat IgG (dilution 1:300; R&D Systems) antibody was used with polyclonal peroxidase labeled anti-goat IgG (H + L; 1:1000; Vector Laboratories Inc., Burlingame, CA, United States); and mouse anti β-actin primary antibody, clone AC-74 (1:5000; Sigma-Aldrich), was used with polyclonal peroxidase labeled anti-mouse IgG (1:2000; Invitrogen). Bands were visualized with the Immobilon Western Chemiluminescence HRP substrate (Merck Millipore). Semiquantitative analysis of band intensities was performed by measuring the area under the peak of plotted lanes with the ImageJ software (National Institutes of Health, Bethesda, MD, United States). For all Western blot quantifications, *n* = 4.

### Luminex

Concentrations of murine IL-4, IL-5, and IL-13, and IL-33 in lung homogenates were analyzed by using Luminex Performance assays (R&D Systems, Abingdon, United Kingdom).

### Periodic Acid–Schiff Staining

Deparaffinized and rehydrated 5-μm lung sections were stained for mucus producing goblet cells by using the periodic acid–Schiff kit (Sigma-Aldrich). Positively stained cells in airway with a perimeter between 800 and 2000 μm were counted using the cell counting tool in ImageJ.

### Total IgE and SplD-Specific IgE ELISA

Total serum IgE was measured with the Mouse IgE Ready-Set-Go! ELISA Kit (affymetrix eBioscience, Vienna, Austria). The SplD-specific IgE ELISA was performed as described previously in detail by coating the plates with 5 μg/mL SplD, and specific IgE was measured in serum.

### Flow Cytometry

The following antibodies were used for identification of immune cells: anti-mouse CD16/CD32 clone 93 (eBioscience), Gr1-FITC (Ly-66) clone RB6-8C5 (eBioscience), SiglecF-PE clone ES22-10D8 (Miltenyi Biotec, Bergisch Gladbach, Germany), CD11b–peridinin-chlorophyll-protein complex (PerCP)–Cy5.5 (eBioscience), F4/80-APC (BD Biosciences), CD8a-PerCp-Cy5.5 clone 53-6.7 (eBioscience), CD4-FITC clone RM4-4 (eBioscience), MHCII clone M5/114.15.2 FITC (eBioscience), CD11c PE-Cy7 clone HL3 (eBioscience), and LIVE/DEAD Fixable Near-IR Dead Cell Stain Kit (Thermo Fisher Scientific). Cells were stained for 30 min at room temperature and washed with PBS; LIVE/DEAD Fixable Near-IR Dead Cell Stain Kit (Thermo Fisher Scientific) was used for 10 min to exclude dead cells. Cells were washed and analyzed by using flow cytometry with the FACS Canto II (BD Biosciences).

### RT-qPCR Analysis of mIL-33 and Serpina3i Expression

For RNA extraction, cells were lysed with RLT buffer (Qiagen), or frozen lung tissues were ground in liquid nitrogen, prior to lysis in RLT (Qiagen). Lysed lung tissues were loaded on the QIAshredder Mini Spin Columns (Qiagen). The RNA was extracted from the cell lysate or QIAshredder Mini Spin Columns eluate following the manufacturer’s instructions of the RNeasy Mini Kit (Qiagen). DNA was digested using the RNase-Free DNase Set (Qiagen). RNA concentrations were measured with Nanodrop, and cDNA was synthesized with the iScript^TM^ Advanced cDNA Synthesis Kit for RT-qPCR (Bio-Rad). For RT-qPCR, *mIL-33*, and *serpina3i* were measured and normalized to *HRPT1* and *RTL32*. The following primers were used (5′-3′ direction):

*HPRT1*-Fwd: CCTAAGATGAGCGCAAGTTGAA; *HPRT1*- Rev: CCACAGGACTAGAACACCTGCTAA; *RTL32*-Fwd: GG CACCAGTCAGACCGATATG; *RTL32*-Rev: CCTTCTCCG CACCCTGTTG; *IL-33*-Fwd: GCTGCGTCTGTTGACACATT; and *IL-33*-Rev: CACCTGGTCTTGCTCTTGGT. Serpina3i primers were purchased from Bio-Rad, intron-spanning (qMmuCID0022819), and *Il1rl1* (encoding ST2) Taqman (QuantiTect Probe PCR Kit; Mm00516117_m1) was purchased from Applied Biosystems. Amplification reactions were performed on the Roche Light Cycler 480. PCR reactions containing 5 ng/well sample cDNA were used with 1 × SsoAdvanced^TM^ Universal SYBR^®^ Green Supermix (Bio-Rad) and 250 nM primer pairs in a final volume of 5 μL. The PCR protocol consisted of 1 cycle at 95°C, 2 min, followed by 44 cycles at 95°C, 5 s; 30 s at 60°C; and 1 s at 72°C. Ct and Tm were calculated by the Roche LightCycler 480 software (Roche Molecular Systems Inc., Pleasanton, CA, United States), and the relative expression of the genes of interest was normalized to the expression of the reference genes *HPRT1* and *RTL32* in qbase^+^ (Biogazelle, Zwijnaarde, Belgium).

### Enzymatic IL-33 Cleavage Assay

One and a half μg of recombinant activated mMCP-4 (0.1 mg/mL in PBS) were incubated alone or with 1.5 μg (1:1 ratio), 7.5 μg (1:5 ratio), or 15 μg (1:10 ratio) of recombinant SERPINA3I (0.1 mg/mL in PBS) for 1 h at 37°C; 12.5 ng of recombinant murine IL-33 (Peprotech, 210-33, Rocky Hill, NJ, United States) in PBS were added to each mixture and incubated again for 1 h at 37°C. Thereafter, samples were denatured (10 min, 95°C with sample buffer) under reducing conditions and loaded on a precast gel (4–15% Mini-PROTEAN TGX Stain-Free Precast Gels, Bio-Rad) to perform SDS-PAGE. IL-33 Western Blot was performed as mentioned above.

### Bioinformatics

Sequence variation data (release v5) from the Mouse Genome Project (33) was used for comparative analyses of the C57BL/6N, CBA, DBA/2, and BALB/c strains relative to the C57BL/6J reference strain. In total, a list of 352 indels affecting the protein coding sequence in at least one of the mouse strains was retrieved using a custom Perl script.

### Statistics

Analysis of data obtained in animal studies was performed using the GraphPad prism version 7 software (GraphPad Software, La Jolla, CA, United States) and one-way ANOVA or Kruskal–Wallis test followed by Tukey multiple comparison or Dunn’s multiple comparison tests, respectively.

## Results

### Lack of Type 2 Airway Inflammation in Repeatedly SplD-Exposed BALB/c Mice

**FIGURE 1:**
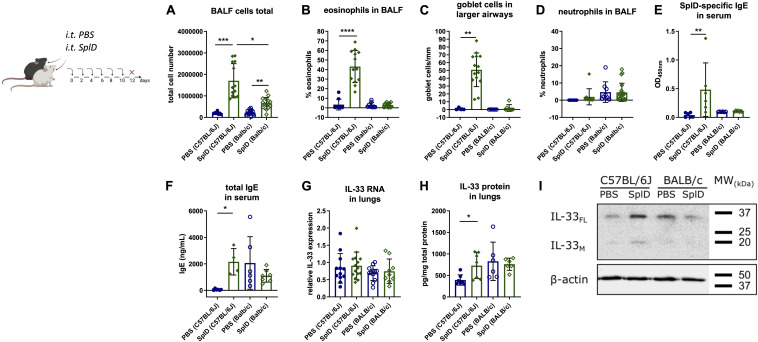
Lack of type 2 airway inflammation and increased IL-33 cleavage in SplD-exposed BALB/c mice. C57BL/6J and BALB/c mice received six i.t. applications of PBS or SplD and 48 h after the last application the samples were collected. **(A)** total cell count, **(B)** percentage of eosinophils, **(C)** Goblet cell count in airways with 800–2000 μm perimeter of periodic acid–Schiff-stained lung sections, and **(D)** neutrophils of bronchoalveolar lavage fluid (BALF). **(E)** SplD-specific and **(F)** total IgE measured by ELISA in sera of mice. **(G)** relative mRNA levels and **(H)** protein levels of IL-33 in lungs determined by RT-qPCR and Luminex, respectively. **(I)** Western blotting of mouse lungs stained for IL-33 and β-actin. One representative blot is shown. Data are presented as mean ± SD. *n* = 4–15. *P < 0.05; **P < 0.01; ***P < 0.001.

C57BL/6J mice respond to repeated intratracheal applications of SplD with key features of allergic asthma (Figure 1A). However, BALB/c mice merely increased total BALF cell numbers (Figure 1A). Eosinophils and neutrophils were nearly absent in SplD-treated BALB/c mice, while C57BL/6J mice showed a strong eosinophilic response toward SplD in the BALF (Figures 1B,C). Goblet cell hyperplasia was not observed in SplD-treated BALB/c mice, in contrast to C57BL/6J mice (Figure 1D). Measurable titers of SplD-specific serum IgE could only be detected in SplD-treated C57BL/6J mice (Figure 1E), and total IgE titers were not different between SplD-sensitized and PBS control BALB/c mice, while a significant up-regulation occurred in the SplD-sensitized C57BL/6J mice (Figure 1F).

Basal and SplD-induced IL-33 mRNA levels, as determined by quantitative RT-qPCR, were similar in the lungs of BALB/c and C57BL/6J mice after 2 weeks of treatment with PBS or SplD (Figure 1G). In contrast, IL-33 protein levels were significantly elevated in SplD-treated C57BL/6J mice but did not change significantly in BALB/c mice compared to the PBS-treated controls (Figure 1H). Less prominent bands of full length (30 kDa; IL-33_FL_) and mature IL-33 (18 kDa; IL-33_M_) was observed by Western blotting in SplD-treated BALB/c mice compared to C57BL/6J mice after 2 weeks of treatment (Figure 1I).

### Diminished Airway Inflammation in BALB/c Mice Is Associated With Faster IL-33 Cleavage Upon a Single Application of SplD

**FIGURE 2:**
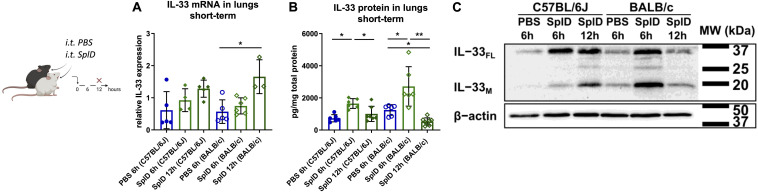
Early degradation of IL-33 in SplD-exposed BALB/c mice. **(A)** mRNA levels and **(B)** protein levels of IL-33 6 and 12 h after PBS or SplD challenge in lungs, determined by RT-qPCR and Luminex, respectively, and **(C)** western blotting of mouse lungs collected 6 and 12 h after a single application of SplD in C57BL/6J or BALB/c mice stained for IL-33 and β-actin. One representative blot is shown. Data are presented as mean ± SD. *n* = 3–8. *P < 0.05; **P < 0.01.

IL-33 expression was measured in the lungs of C57BL/6J and BALB/c mice 6 and 12 h after a single i.t. application of SplD. 6 h after SplD exposure, the IL-33 mRNA levels were not significantly increased and were comparable between C57BL/6J and BALB/c mice. However, 12 h after SplD exposure, significantly higher IL-33 mRNA levels were observed in BALB/c mice, while in C57BL/6J mice a statistically insignificant trend to increased IL-33 mRNA levels was observed (Figure 2A). In contrast to mRNA expression, IL-33 protein levels were significantly up-regulated in both mouse strains 6 h after a single i.t. application of SplD. The levels were significantly decreased 12 h after SplD application in both mouse strains compared to 6 h after SplD exposure (Figure 2B). When comparing full length IL-33_FL_ of BALB/c and C57BL/6J mice after 6 and 12 h of a single application of SplD using western blotting analysis, there was a strong increase of IL-33_FL_ in both strains, but in BALB/c mice, the proteolytic processing of IL-33_FL_ and IL-33_M_ was accelerated (Figure 2C). In BALB/c mice, the bands representing IL-33_ FL_ and IL-33_M_ were most pronounced after 6 h and declined 12 h after SplD treatment. On the contrary, IL-33_FL_ levels were comparable after 6 and 12 h of SplD treatment in C57BL/6J mice, and IL-33_M_ was increased only after 12 h (Figure 2C).

Taken together, the lower IL-33 protein amounts in BALB/c mice at 12 h after a single application and 48 h after repeated applications could be indicative of an increased activity protease cleaving IL-33 in the lungs of BALB/c.

### Local Administration of Recombinant IL-33 in SplD-Treated BALB/c Mice Restores the Allergic Response

**FIGURE 3:**
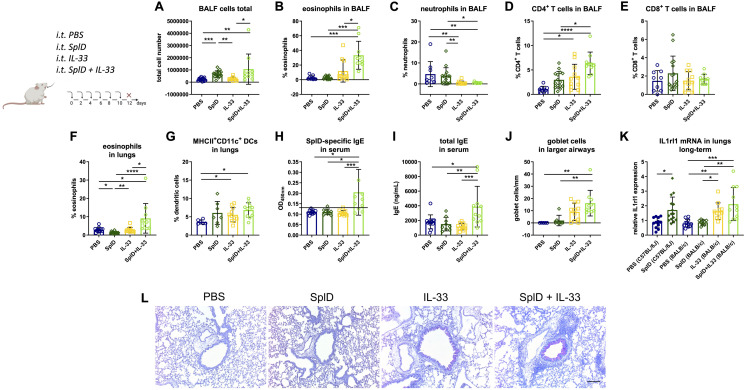
Supplementation with IL-33 restores the asthmatic response and has a synergistic effect on SplD-induced allergic sensitization in BALB/c mice. BALB/c wild-type mice were treated with six i.t. applications of PBS, SplD, IL-33 or SplD + IL-33 every second day. 48 h after the last application, mice were euthanized, and samples were collected. **(A)** Total cell count and percentage of **(B)** eosinophils, **(C)** neutrophils, **(D)** CD4 + T cells, and **(E)** CD8 + T cells in the BALF were determined by flow cytometry. Percentage of **(F)** eosinophils and **(G)** MHCII + CD11c + dendritic cells (DCs) in the lungs were determined by flow cytometry. **(H)** SplD-specific IgE and **(I)** total IgE in sera of mice, measured by ELISA; the black line indicates the background optical density. **(J)** Goblet cell count in airways of 800–2000-μm perimeter in periodic acid–Schiff-stained lung sections. **(K)** Relative *Il1rl1* mRNA expression in lungs. **(L)** representative images of periodic acid–Schiff-stained lung sections. Data are presented as mean ± SD. *n* = 4–15. *P < 0.05; **P < 0.01; ***P < 0.001.

We aimed to compensate the lack of IL-33 in BALB/c mice by intratracheal applications of exogenous IL-33 directly after each SplD application. After the addition of IL-33 to SplD, the percentage of eosinophils in the BALF was significantly elevated, while the percentage of neutrophils was decreased without major effect on the total cell number in BALF (Figures 3A–C). The combined treatment with SplD + IL-33 induced an up-regulation of CD4^+^ T cells in the BALF, compared to the SplD or IL-33 only treatment groups (Figure 3D). The numbers of CD8^+^ cells in the BALF did not significantly differ between the treatment groups (Figure 3E). In the lungs, the percentage of eosinophils was significantly elevated in mice that received SplD + IL-33 compared to all control groups (Figure 3F). BALB/c mice treated with IL-33 alone or in combination with SplD showed elevated percentages of MHCII^+^CD11c^+^ dendritic cells in the lungs (Figure 3G). The levels of IL-5 were below detection levels in the lungs of BALB/c mice treated with SplD, and no up-regulation could be seen after treatment with IL-33 or a combination of IL-33 and SplD (Supplementary Figure 2B). The levels of IL-4 and IL-13 also remained very low in all experimental groups without significant difference compared between the groups (Supplementary Figures 2A,C). SplD-specific serum IgE (Figure 3H) and total serum IgE (Figure 3I) were significantly up-regulated in the SplD + IL-33 group. Thus, the addition of exogenous IL-33 could sensitize BALB/c mice to SplD, while SplD given alone did not stimulate IgE production in these mice. Goblet cell hyperplasia was observed in the IL-33 only and the SplD + IL-33 groups, indicating that goblet cell formation and mucus production are strongly dependent on IL-33 (Figures 3J,L). IL-33 supplementation restored the up-regulation of *Il1rl1* mRNA in BALB/c mice to a comparable degree as in C57BL/6J mice, suggesting that the rapid cleavage of IL-33 explains the absence of ST2 induction in BALB/c mice that were treated with SplD alone (Figure 3K).

### CBA and DBA/2 Mice Present a Diminished Type 2 Response Toward SplD While C57BL/6N Mice Are Identified as SplD Responders

**FIGURE 4:**
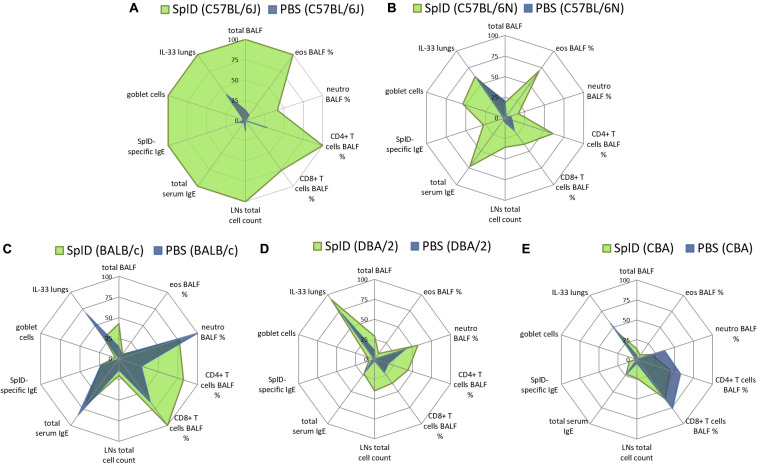
C57BL/6J and C57BL/6N mice respond to SplD with key features of allergic asthma, while BALB/c, CBA, and DBA/2 mice lack a clear type 2 response. Radar graphs, presenting relative values of measured parameters from **(A)** C57BL/6J, **(B)** C57BL/6N, **(C)** BALB/c, **(D)** DBA/2, and **(E)** CBA mice treated repeatedly with PBS or SplD. Per parameter, the highest mean value between the groups was set at one hundred percent.

C57BL/6N, CBA, and DBA/2 mice were tested in addition to C57BL/6J and BALB/c mice to define SplD responders and non-responders in order to facilitate the search for the genetic cause underlying the different inflammatory responses toward SplD. We compared the relative magnitude of inflammatory response parameters in different mouse strains and presented the data in radar graphs where the highest mean value of each parameter amongst all five tested strains was taken as one hundred percent (Figure 4A). SplD-treated C57BL/6J mice showed the strongest response in all measured parameters, which indicate a Th2-biased inflammation, but not in those reflecting their neutrophil and CD8^+^ T cell responses (Figure 4A). C57BL/6N mice show a comparable reaction pattern as C57BL/6J mice upon SplD treatment, even though the magnitude of response was significantly reduced and the total cell numbers in BALF cells were not increased (Figure 4B). BALB/c mice did not develop a Th2 inflammation in response to SplD. There was, however, an increase in CD8^+^ T cells, but as shown in Figure 3E, the numbers of CD8^+^ T cells in the BALF were low (Figure 4C). DBA/2 mice presented with a very weak response toward SplD, which was, however, neutrophilic rather than eosinophilic (Figure 4D). CBA mice responded in a manner comparable to DBA/2 mice, but neutrophil and T cell counts were lower than in the PBS control group of this strain (Figure 4E). Goblet cell hyperplasia was only found in SplD-treated C57BL/6J and C57BL/6N mice (Figure 4). The data of each measured parameter of C57BL/6N, DBA/2, and CBA mice with the statistical analysis can be found in Supplementary Figure 1. We classified BALB/c, CBA, and DBA/2 mice as SplD non-responder strains due to the weak or absent type 2 inflammatory response in comparison to C57BL/6J and C57BL/6N mice, which were defined as SplD-responder strains.

### Genome Wide Comparison of SplD Responder and Non-responder Strains

**FIGURE 5:**
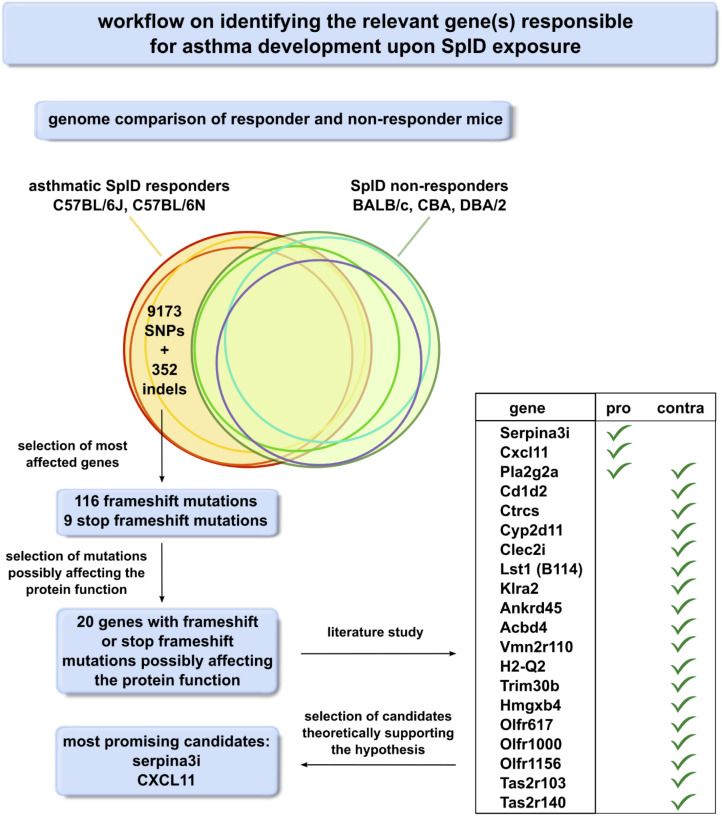
Workflow scheme for the identification of the relevant gene(s) responsible for asthma development upon SplD exposure. Genomes of SplD responders (C57BL/6J and C57BL/6N mouse strains) were compared to the genomes of SplD non-responders (BALB/c, CBA and DBA/2 mouse strains). The indels specific for SplD responders were further analyzed for their consequence on the gene product, and the frameshift mutations were selected as the mutations affecting protein function the most.

Sequence variation data (release v5) from the Mouse Genome Pro (33) was used for comparative analyses of the C57BL/6N, CBA, DBA/2, and BALB/c strains relative to the C57BL/6J reference strain. In total, a list of 352 indels affecting the protein coding sequence in at least one of the mouse strains was retrieved using a custom Perl script. Out of this list, 116 frameshift mutations and nine stop-codon-inducing frameshift mutations were selected for further analysis as these mutations are expected to exert the most severe effects on the function of the encoded proteins. These variant consequences, predicted by the Ensembl Variant Effect Predictor (VEP), were further investigated using CLC Main Workbench to predict if the main transcript is affected and if the frameshift possibly affects the function of the mutated gene product. In 20 genes, frameshift or stop frameshift mutations were identified upstream or in their active domain. These 20 genes were studied by literature research to find pro- and contra-arguments for them being crucial for the asthmatic response toward SplD (Supplementary Table 1). Our workflow (Figure 5) allowed us to identify *Serpina3i* and *CXCL-11* as the most promising candidates. CXCL-11 mRNA was not detectable by RT-qPCR in the lungs of SplD-treated mice in any of the five mouse strains (data not shown) and was therefore excluded.

### SERPINA3I—Homology Model

**FIGURE 6:**
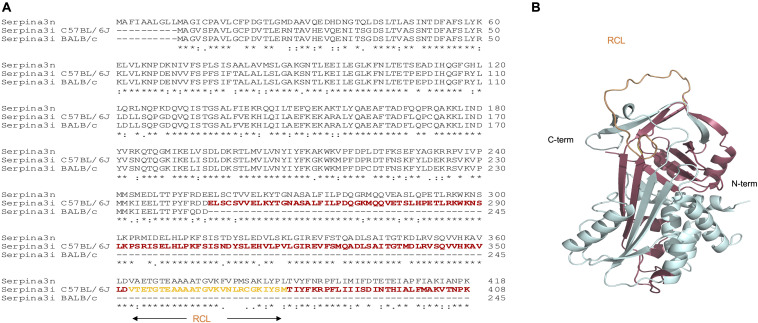
Protein sequence alignment of murine SERPINA3N and SERPINA3I from C57BL/6J and BALB/c mice and structural mapping of SERPINA3I deficiency in BALB/c mice. **(A)** Alignment of the sequences of murine SERPINA3N (Uniprot sequence Q91WP6) and the sequences for SERPINA3I from mouse strains C57BL/6J and BALB/c. The sequence segment absent in BALB/c SERPINA3I as a result of the frameshift mutation at Cys249 and the introduction of a stop codon after residues 251 is colored in red. The reactive center loop (RCL) locates within the deleted sequence and is colored in orange. **(B)** Cartoon representation of a predicted model for murine SERPINA3I based on the crystal structure of murine SERPINA3N (Horvath et al. 2005) as computed by the program RaptorX (Källberg et al. 2012). The figure was produced with PyMOL v. 2.3.0.

Mice harbor an unusually large group of *Serpina3* genes as a result of extensive gene duplication and diversification, which in turn encode for 13 closely related SERPINA3 protein products, albeit with differing target specificities and biodistribution. The high level of sequence similarity shared by murine SERPINA3 proteins essentially guarantees a highly conserved core structure. To this end, murine SERPINA3I displays a 74% sequence identity to SERPINA3N (Figure 6A) for which the high resolution crystal structure is available. We harnessed the high sequence homology of the two proteins and the available structural information to construct a homology model for SERPINA3I (Figure 6B). Thus, SERPINA3I is expected to adopt a fold displaying a main central beta-sandwich consisting of two beta-sheets flanked by a cluster of 7–9 alfa helices. This sets the structural stage for a third beta-sheet at the top of the fold to help project the flexible RCL. The RCL contains an enzyme cleavage site (P1-P1’), which is located at the C-terminus of the protein. RCL is expected to be cleaved by a target protease at P1 and P1’, leading to its covalent attachment via the main carbonyl carbon of the P1 residue of the SERPINA3I. This would be expected to establish a stable complex between SERPINA3I and a protease, such as mMCP-4 leading to the inhibition of mMCP-4. The frameshift mutation in serpina3i was stated in the sanger mouse genome sequencing database (rs242560633). The sequence lacks a cytosine at bp747, which results in a frameshift at residue Cys249 and a stop codon after residue 251. As the RCL starts at G357 (31), the mutated *Serpina3i* is predicted to result in an unfunctional protein. In C57BL/6J and C57BL/6N mice, SERPINA3I is predicted to be full length, comprising 408 amino acids (NP_001186869.1).

**FIGURE 7:**
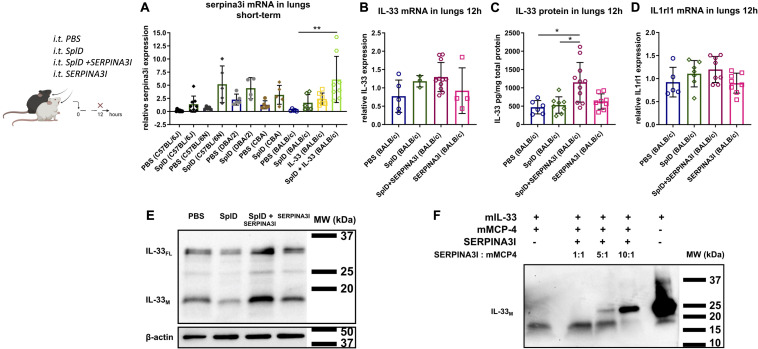
*Serpina3i* mRNA expression is up-regulated in mouse lungs upon SplD and IL-33 exposure and decelerates IL-33 degradation *in vivo* and *in vitro*. **(A)** relative mRNA expression of *Serpina3i* in lungs 6 h after a single i.t. application of PBS or SplD in C57BL/6J, C57BL/6N, DBA/2, CBA, and BALB/c mice as well as BALB/c mice 6 h after IL-33 or SplD + IL-33 i.t. treatment determined by RT-qPCR. Relative mRNA levels determined by RT-qPCR of IL-33 **(B)** and *Il1rl1* **(D)** determined by RT-PCR. IL-33 protein determined by **(C)** Luminex and **(E)** Western blot in mouse lungs of BALB/c mice 12 h after PBS, SplD, SERPINA3I, or SplD + SERPINA3I treatment. Data are presented as mean ± SD. *n* = 5–9. **(F)** IL-33 Western blot of recombinant mature murine IL-33 (20 kDa) co-incubated with mMCP-4 for 1 h with and without 1 h preincubation with increasing concentrations of SERPINA3I. One representative blot of three independent experiments is shown. *P < 0.05; **P < 0.01.

To obtain insights into the structural and functional consequences of the identified frameshift mutation, we compared the sequences of full-length and truncated SERPINA3I to the closely related SERPINA3N (Figure 6A) and constructed a homology model of SERPINA3I based on the crystal structure of the mouse SERPINA3N (34) (Figure 6B). Our analyses illustrate that the predicted protein product of *Serpina3i* in mouse strain BALB/c would display a debilitating sequence truncation from Cys259 onward that includes the RCL and would prevent the protein from folding to a functional form (Figures 6A,B). Thus, the observed deletion would ultimately cause deficiency in SERPINA3I in BALB/c mice and other mouse strains bearing the same mutation. A public online tool^1^ is available to analyze different mouse strains having the same *Serpina3i* frameshift mutation (35). Gene expression of *Serpina3i* in mouse lungs collected 6 h after a single i.t. application of PBS or SplD showed no significant up-regulation in SplD-treated mice. Furthermore, *Serpina3i* mRNA was up-regulated in response to IL-33 + SplD treatment in BALB/c mice suggesting a possible involvement of SERPINA3I in IL-33 signaling or regulation (Figure 7A). In other mouse strains, no difference in gene expression of *Serpina3i* after a single i.t. application SplD was observed. Importantly, the observed difference in *Serpina3i* gene expression difference does not fully reflect the observed phenotypic difference since a truncation of the gene in the non-responder mouse strains BALB/c, CBA, and DBA/2 would result in an unfunctional protein.

### SERPINA3I Decelerates IL-33 Degradation via mMCP-4

To test the functional activity *in vivo*, recombinant full-length SERPINA3I was administered intratracheally directly after SplD to BALB/c mice. SERPINA3I treatment did not cause any up-regulation of IL-4, IL-5, or IL-13 in the lungs of BALB/c mice (Supplementary Figures 2D–F), while, IL-33_FL_ and IL-33_M_ protein levels were significantly higher in the lungs of mice receiving SplD + SERPINA3I than when SplD was administered alone (Figure 7C). As expected the *IL-33* and *Il1rl1* mRNA expression levels were not significantly changed (Figures 7B,D). The analysis of the cleavage bands of IL-33 by Western blot revealed that both IL-33_FL_ and IL-33_M_ were increased in SplD + SERPINA3I-treated mice (Figure 7E). These results indicate that SERPINA3I can decelerate the degradation of IL-33_FL_ that was induced by *in vivo* application of SplD. Moreover, preincubation of mMCP-4 with SERPINA3I prevents mMCP-4-induced enzymatic degradation of recombinant murine IL-33_M_ in a dose-dependent manner (Figure 7F).

## Discussion

In this study, we have demonstrated that the allergic response toward the *S. aureus* protease SplD is mouse strain dependent. Our data suggest that rather than differences in *S. aureus* strains, the genetic variability of the host could be an important determinant of the immune response to Spls in human *S. aureus* carriers. In humans that are naturally exposed to *S. aureus*, the quality of the Spl-specific antibody and T-cell response is strongly biased toward a Th2 profile on average with extensive variation between individuals (3). This could partially be explained by differences in exposure to Spls, which are encoded in around 80% of clinical *S. aureus* isolates. Our data, however, suggest that genetic differences affecting the posttranslational IL-33 regulation could also play a role.

**FIGURE 8:**
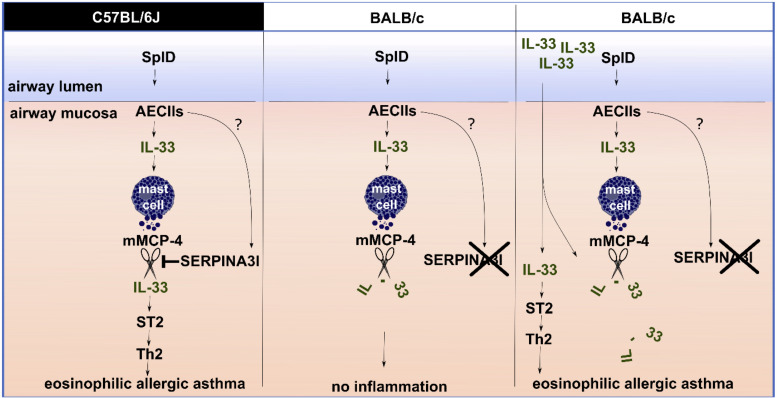
The role of SERPINA3I in the IL-33 regulation mechanisms in C57BL/6J and BALB/c mice upon SplD exposure. SplD administration to the murine airway mucosa causes IL-33 release from alveolar epithelial cells type 2 (AECIIs), and *Serpina3i* up-regulation in the lungs. In the lungs, IL-33 triggers mast cells to release murine mast cell protease 4 (mMCP-4), which can degrade IL-33. In C57BL/6J mice, functional SERPINA3I is present; it inhibits mMCP-4 and thereby prevents IL-33 degradation (shown *in vitro*). As a result, IL-33 triggers a type 2 inflammatory response via the IL-33/ST2 axis. BALB/c mice lack a functional SERPINA3I protein due to a mutation. Consequently, SplD-triggered IL-33 is degraded, and no type 2 inflammation is triggered. In BALB/c mice the administration of an active form of recombinant IL-33 in excess additionally to SplD is leading to an inflammatory response comparable to C57BL/6J mice since the degradation of IL-33 can be circumvented to the degree that the IL-33/ST2 signaling can take place.

While C57BL/6J and C57BL/6N mice show typical features of allergic asthma, this response is lacking or significantly diminished in BALB/c, CBA, and DBA/2 mouse strains. C57BL/6 and BALB/c mice have often been referred to as Th1-prone or Th2-prone strains, respectively (36). In striking contrast, C57BL/6 mice presented a higher eosinophilic Th2-biased response than BALB/c mice in our SplD sensitization model. A stronger eosinophilic response in C57BL/6 mice compared to BALB/c mice has been described previously in other murine asthma models using OVA/alum, house dust mite extract or the house dust mite allergen Der p 1 (37–40). In the mentioned models, however, BALB/c mice still developed a weak eosinophilic response, which might be due to the activation of other pathways next to the IL-33/ST2 signaling axis. We have shown previously that the type 2 inflammatory response toward SplD and lung infiltration with IL-13^+^ type innate lymphoid cells and IL13^+^ CD4^+^ T cells in C57BL/6J mice is mainly mediated by IL-33 (4). In this study, we found that C57BL/6J and BALB/c mice also differ in their post-translational regulation of IL-33. A single intratracheal application of SplD caused an up-regulation of IL-33 in both mouse strains after 6 h; however, IL-33 was degraded more rapidly in BALB/c than in C57BL/6J mice. In line with these results, C57BL/6 mice responded to long-term exposure to SplD with significantly increased *Il1rl1* expression, while this was not the case in BALB/c mice. We excluded an impairment in ST2 expression downstream of IL-33 in BALB/c mice, because the administration of IL-33 restored the up-regulation of *Il1rl1* and, in combination with SplD, led to a strong eosinophilic response in BALB/c mice. Our observed mouse strain dependence of the phenotype in the SplD asthma model illustrates the importance of the genetic background when testing a potential allergen in mice (Figure 8). The observed loss of function mutation in *Serpina3i* could also explain other mouse strain-dependent differences in allergic asthma, which have previously been reported. Genetic differences in the regulation of IL-33 could be determinants of allergic sensitization or severity of airway diseases. In humans there are several single-nucleotide polymorphisms (SNPs) upstream of the IL-33 gene, which are associated with asthma prevalence (41); however, the functional consequences of these SNPs is largely unknown. The human *SERPINA3* gene has expanded by gene duplication into a cluster of 14 equivalent mouse *SERPINA3 genes, Serpina3a-n*. Based on protein expression patterns, especially in the brain, *Serpina3n* is considered as the murine orthologue of the human *SERPINA3* (31, 34). The murine SERPINA3N is known to inhibit leukocyte elastase and cathepsin G (34), but the mast cell chymase inhibitory function of α-1 anti-chymotrypsin, however, could potentially be conserved in SERPINA3I and could explain our observed mechanism. Mutations in SERPINA3 have been described to have an impact in lung diseases. An association between childhood onset asthma and a 1.4 kb gain mutation downstream of *SERPINA3* has been reported (42). Mutations in *SERPINA3* leading to decreased α-1 anti-chymotrypsin serum levels were associated with the inflammatory lung diseases COPD and emphysema (43). Our study revealed a novel IL-33 regulation pathway of SERPINA3I inhibiting the IL-33 degrading mast cell chymase mMCP-4, which could be, with the equivalent human proteins, of general importance in allergic airway diseases (44). The human *SERPINA3* is a known mast cell chymase inhibitor and could thus be relevant in IL-33 regulation (45).

The study of individual differences in this protease-serpin interaction network may help to identify predictive biomarkers for Th2-biased airway disease development, especially in individuals colonized by *S. aureus*.

## Data Availability Statement

The raw data supporting the conclusions of this article will be made available by the authors, without undue reservation.

## Ethics Statement

The animal study was reviewed and approved by Ethics committee of Faculty of Medicine and Health Sciences, Ghent University.

## Author Contributions

AT designed and performed experiments, analyzed data, and wrote the original draft. SV performed experiments. PH performed genome-wide comparison analysis and predicted protein function affecting frameshift mutations. SS performed protein sequence and structural analyses. MN provided SplD. GH and SA performed experiments. ND performed experiments. SD performed experiments. LH provided recombinant Mcpt-4. WD and PV provided reagents. DK provided analysis tools and supervised experiments. BB provided reagents. CB supervised experiments and edited the manuscript. OK designed, performed, supervised, and analyzed experiments, and edited the manuscript. All authors discussed the results and commented on the manuscript.

## Conflict of Interest

The authors declare that the research was conducted in the absence of any commercial or financial relationships that could be construed as a potential conflict of interest.

## Footnotes

1 http://bioit2.irc.ugent.be/prx/mousepost/Home.php

## Supplementary Material

Supplementary Figure 1 Inflammatory profile of C57BL/6N, DBA/2 and CBA mice upon repeated intratracheal applications of PBS or SplD for 2 weeks. **(A)** Total cell count, **(B)** percentage of eosinophils, **(C)** neutrophils, **(D)** CD4^+^ T cells, and **(E)** CD8 + T cells in the BALF analyzed by flow cytometry. **(F)** Total cell count of local draining lymph nodes, **(G)** total, and **(H)** SplD-specific IgE in serum measured by ELISA. **(I)** Goblet cells in airways with a perimeter of 800–2000 μm, analyzed in periodic acid–Schiff-stained lungs. **(J)** IL-33 protein levels in lungs measured by Luminex. **(K)** Relative Il1rl1 mRNA expression in lungs. Data are presented as mean ± SD. *n* = 4–6.

Supplementary Figure 2 Analysis of type-2 cytokines in the lung homogenates of mice by Luminex. The levels of IL-4 **(A)**, IL-5 **(B)**, and IL-13 **(C)** cytokines in the lungs of C57BL/6J, and BALB/c mice treated with six i.t. applications of PBS, SplD, IL-33, or SplD + IL-33 every second day for 2 weeks. The lung homogenates of BALB/c mice after 12 h after a single i.t. application of PBS, SplD, SERPINA3I, or SplD + SERPINA3I treatment were analyzed for IL-4 **(D)**, IL-5 **(E)**, and IL-13 **(F)**. Data are presented as mean ± SD. *n* = 6. The limit of detection of each cytokine is indicated by a dotted line.

Supplementary Table 1 A list of mutated genes between SplD-responder and non-responder strains. Mutation, predicted consequence for the protein function, pro- and contra arguments for being a candidate gene responsible for asthma development according to literature research.

Supplementary Table 2 A summary of experimental procedures per figure.
