# Supplementary figures and images for: Mouse Strain-Dependent Difference Toward the Staphylococcus aureus Allergen Serine Protease-Like Protein D Reveals a Novel Regulator of IL-33
Source: Front Immunol. 2020 Sep 25;11:582044. doi: 10.3389/fimmu.2020.582044 (PMC7544847; doi:10.3389/fimmu.2020.582044)

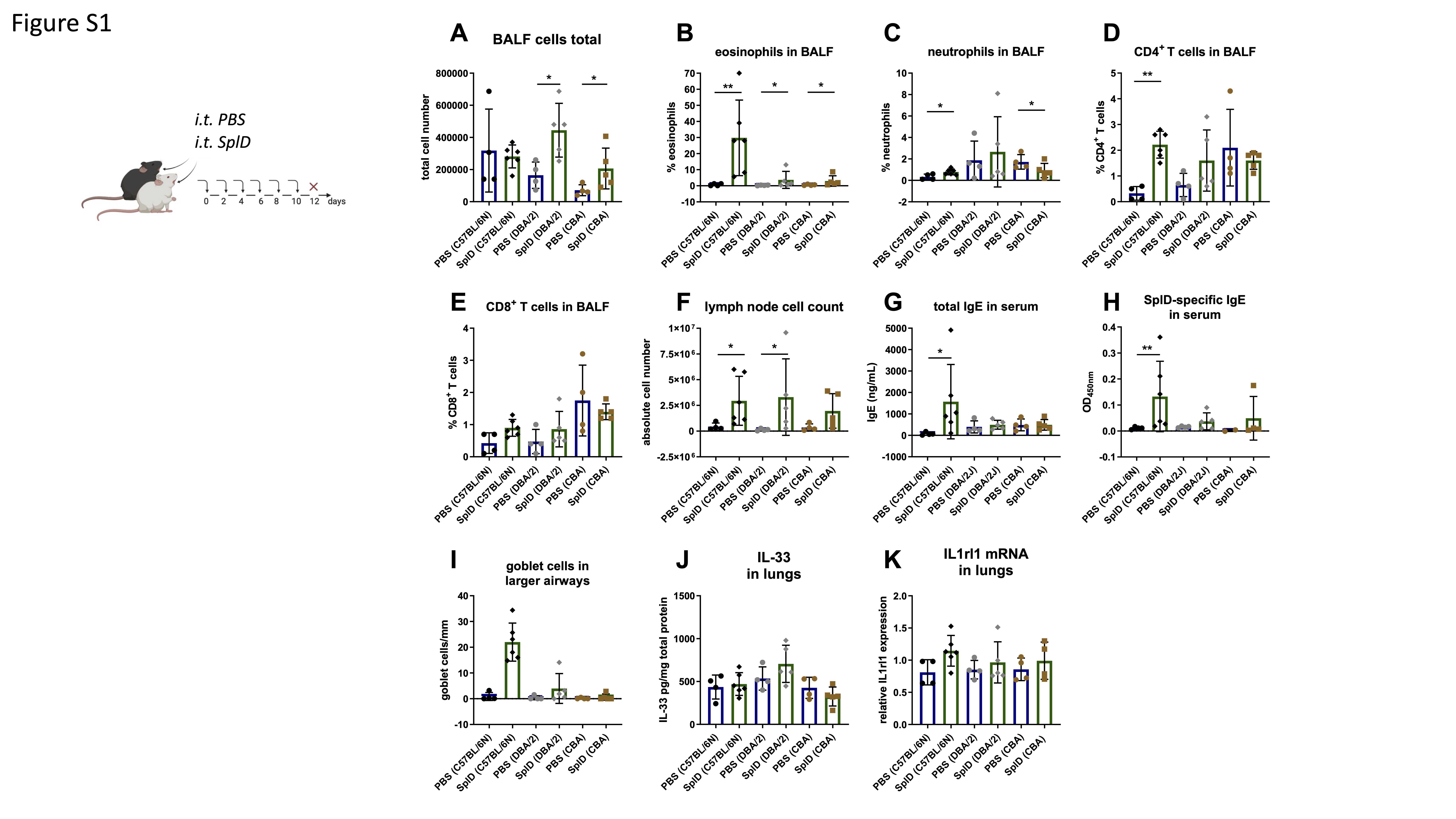

Supplement: Supplementary Figure 1 — Inflammatory profile of C57BL/6N, DBA/2 and CBA mice upon repeated intratracheal applications of PBS or SplD for 2 weeks. (A) Total cell count, (B) percentage of eosinophils, (C) neutrophils, (D) CD4+ T cells, and (E) CD8 + T cells in the BALF analyzed by flow cytometry. (F) Total cell count of local draining lymph nodes, (G) total, and (H) SplD-specific IgE in serum measured by ELISA. (I) Goblet cells in airways with a perimeter of 800–2000 μm, analyzed in periodic acid–Schiff-stained lungs. (J) IL-33 protein levels in lungs measured by Luminex. (K) Relative Il1rl1 mRNA expression in lungs. Data are presented as mean ± SD. n = 4–6. [file Image_1.JPEG]

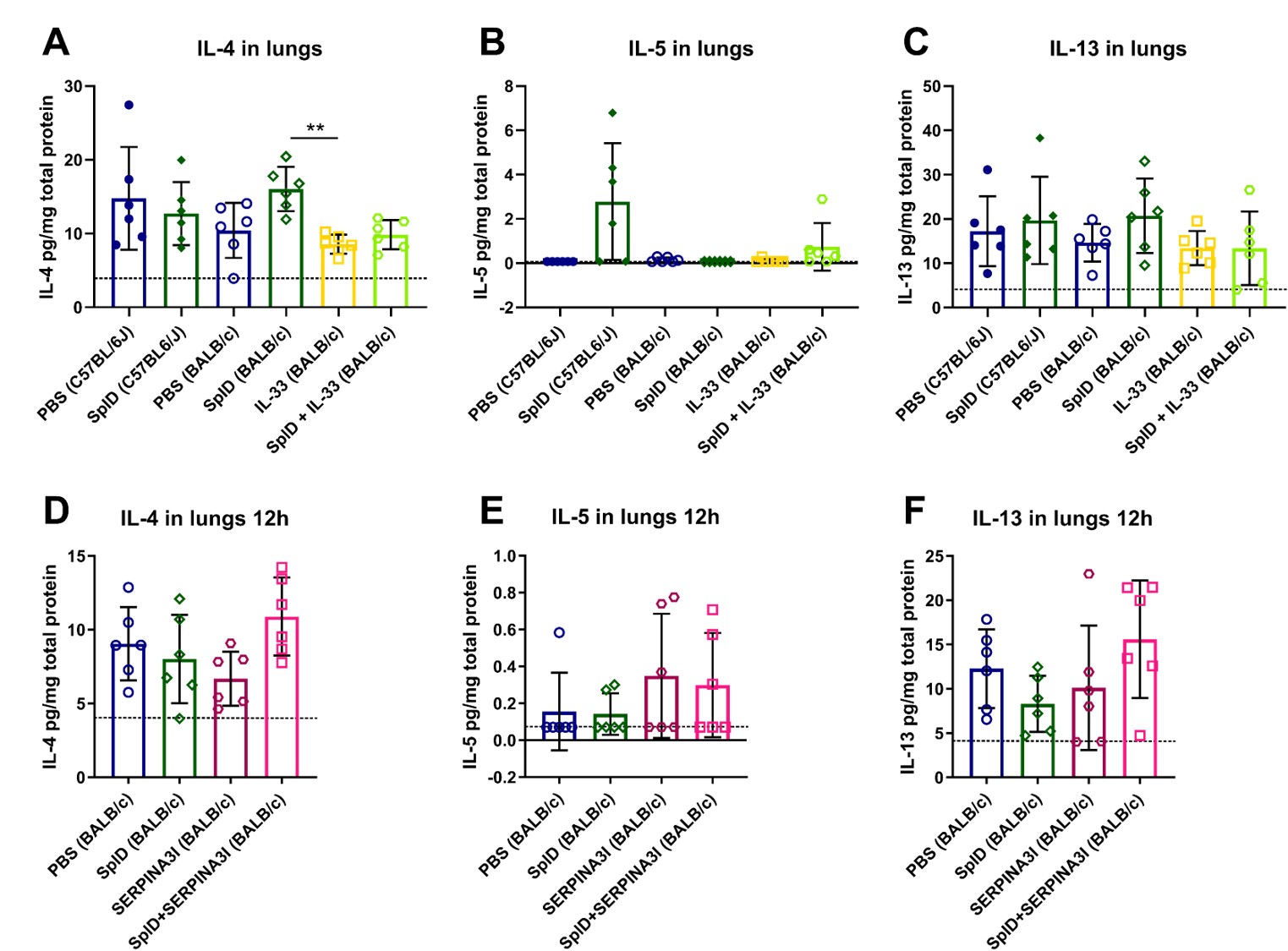

Supplement: Supplementary Figure 2 — Analysis of type-2 cytokines in the lung homogenates of mice by Luminex. The levels of IL-4 (A), IL-5 (B), and IL-13 (C) cytokines in the lungs of C57BL/6J, and BALB/c mice treated with six i.t. applications of PBS, SplD, IL-33, or SplD + IL-33 every second day for 2 weeks. The lung homogenates of BALB/c mice after 12 h after a single i.t. application of PBS, SplD, SERPINA3I, or SplD + SERPINA3I treatment were analyzed for IL-4 (D), IL-5 (E), and IL-13 (F). Data are presented as mean ± SD. n = 6. The limit of detection of each cytokine is indicated by a dotted line. [file Image_2.JPEG]
